# RAMP2 Influences Glucagon Receptor Pharmacology via Trafficking and Signaling

**DOI:** 10.1210/en.2016-1755

**Published:** 2017-06-06

**Authors:** Jaimini Cegla, Ben J. Jones, James V. Gardiner, David J. Hodson, Thomas Marjot, Emma R. McGlone, Tricia M. Tan, Stephen R. Bloom

**Affiliations:** 1 Division of Diabetes, Endocrinology and Metabolism, Imperial College London W12 0NN, United Kingdom; 2 Institute of Metabolism and Systems Research, University of Birmingham, Edgbaston B15 2TT, United Kingdom; 3 Centre for Endocrinology, Diabetes and Metabolism, Birmingham Health Partners, Birmingham B15 2TH, United Kingdom; 4 Centre of Membrane Proteins and Receptors, University of Birmingham, Birmingham B15 2TT, and University of Nottingham, Nottingham NG7 2RD, United Kingdom

## Abstract

Endogenous satiety hormones provide an attractive target for obesity drugs. Glucagon causes weight loss by reducing food intake and increasing energy expenditure. To further understand the cellular mechanisms by which glucagon and related ligands activate the glucagon receptor (GCGR), we investigated the interaction of the GCGR with receptor activity modifying protein (RAMP)2, a member of the family of receptor activity modifying proteins. We used a combination of competition binding experiments, cell surface enzyme-linked immunosorbent assay, functional assays assessing the G*α*s and G*α*q pathways and *β*-arrestin recruitment, and small interfering RNA knockdown to examine the effect of RAMP2 on the GCGR. Ligands tested were glucagon; glucagonlike peptide-1 (GLP-1); oxyntomodulin; and analog G(X), a GLP-1/glucagon coagonist developed in-house. Confocal microscopy was used to assess whether RAMP2 affects the subcellular distribution of GCGR. Here we demonstrate that coexpression of RAMP2 and the GCGR results in reduced cell surface expression of the GCGR. This was confirmed by confocal microscopy, which demonstrated that RAMP2 colocalizes with the GCGR and causes significant GCGR cellular redistribution. Furthermore, the presence of RAMP2 influences signaling through the G*α*s and G*α*q pathways, as well as recruitment of *β*-arrestin. This work suggests that RAMP2 may modify the agonist activity and trafficking of the GCGR, with potential relevance to production of new peptide analogs with selective agonist activities.

## Teaser

A combination of competition binding, functional assays assessing the G*α*s and Gq pathways and *β*-arrestin recruitment, and siRNA knockdown was used to examine the effect of RAMP2 on the GCGR.

Gut and pancreatic hormones involved in appetite regulation are an attractive target for the development of drugs that aim to cause effective weight loss with minimal side effects. Glucagon potently increases satiety and acutely reduces food intake in humans (1). It also significantly increases energy expenditure in humans (2–4). This, in association with the anorectic effects of glucagon (1), enhances its usefulness as an antiobesity therapy.

The glucagon receptor (GCGR) is a 7-transmembrane class B G protein–coupled receptor (GPCR). It classically activates adenylyl cyclase through G*α*s with subsequent activation of protein kinase A signaling (5, 6). In hepatocytes, elevated protein kinase A activity suppresses glycolysis and glycogen synthesis and enhances gluconeogenesis and glycogenolysis (7, 8). However, activation of GCGR also stimulates the phospholipase C–inositol phosphate pathway in hepatocytes via G*α*q, inducing intracellular calcium (Ca^2+^) signaling and stimulating glycogenolysis and gluconeogenesis (6, 9). Although work to unpick glucagon signaling pathways has been underway since the 1970s, it has focused primarily on understanding the interactions involved in the downstream effects in the liver and the pancreas. Less attention has been paid to the role of specific pathways in the extrahepatic roles of glucagon, namely in appetite regulation and control of energy expenditure. As a prototypical class B GPCR, the GCGR is desensitized and sequestered in the cytosol following activation (10–12). The internalized receptor is then either recycled to the cell surface or targeted for degradation. Krilov *et al.* (13, 14) recently demonstrated that the GCGR recycles to the plasma membrane in a *β*-arrestin–dependent manner and that downregulation of *β*-arrestins significantly reduces recycling.

Understanding the interaction of these pathways may allow "biasing" of signaling to exploit desirable downstream effects (15, 16). A particularly well-characterized example of an accessory protein that clearly alters the pharmacology of GPCRs is a family of single transmembrane proteins known as receptor activity modifying proteins (RAMPs). RAMPs were discovered as proteins that interact with the calcitonin receptor–like receptor and calcitonin receptor to give rise to receptors for different ligands (17). These four ligands (calcitonin, amylin, calcitonin gene-related peptide, and adrenomedullin) bind to two receptors and in the presence of the three RAMPs give rise to seven different receptor types with distinct pharmacology (18). Additionally, RAMPs have a role in receptor trafficking, including translocation from the endoplasmic reticulum to the Golgi, internalization, and recycling of the receptor (19–26). RAMPs heterodimerize with several class B and C GPCRs and influence their function and life cycle (27, 28). The ability of RAMPs to influence downstream signaling pathways is an exciting concept because it may enable the creation of biased agonists that fully exploit the therapeutic potential of clinically important receptors.

The functional impact of RAMPs on GCGR pharmacology is not clearly understood. More than 10 years ago, the Christopoulos group (27) showed that the GCGR may interact with RAMP2. Recently, one study found that RAMP2 may alter GCGR ligand selectivity and G protein preference using yeast reporter systems (29). The work presented here is concerned with further understanding the effect of RAMP2 on the pharmacology of the GCGR in mammalian cells.

## Materials and Methods

### Peptides

Human GCG, glucagonlike peptide-1 (GLP-1), and oxyntomodulin were purchased from Bachem Ltd. (St. Helens, United Kingdom). GLP-1(7-36)NH_2_ was the form used in all experiments, and is hereafter referred to simply as GLP-1. A dual glucagon/GLP-1 analog, G(X), was designed in the Department of Investigative Medicine, Imperial College London, and custom synthesized by using solid-phase peptide synthesis (Bachem Ltd.). G(X) contains identical amino acid sequences to glucagon from positions 1 to 15 as the *N*-terminal of glucagon is critical for glucagon receptor binding and activation (30). To create a dual agonist that is also effective at the GLP-1 receptor, G(X) has been modified to resemble exendin-4. This peptide, first isolated from the venom of the lizard *Heloderma* species, is a potent agonist at the human GLP-1 receptor (31, 32). Also favorable is its prolonged pharmacokinetic profile compared with native GLP-1. Therefore, from positions 16 to 34, amino acid substitutions have been made to resemble exendin-4.

### Establishing a cellular coexpression system for RAMP2 and GCGR

Chinese hamster ovarian (CHO-K1 cells; GeneBLAzer GCGR-CRE-*bla* CHO-K1 cells, K1855A; Invitrogen, Carlsbad, CA) cells expressing the GCGR were cultured in Dulbecco’s modified Eagle medium (DMEM) supplemented with 10% fetal bovine serum, 0.1 mM nonessential amino acids, 25 mM HEPES (pH, 7.3), 100 IU/mL penicillin, 100 μg/mL streptomycin, and 5 μg/mL blastocidin. This cell line expressed no background RAMP2, as confirmed by using quantitative polymerase chain (qPCR) reaction (threshold cycle values > 32). The human RAMP2 DNA construct (pCMV6-AC-RAMP2) (Origene, Rockville, MD) was transfected into CHO-K1 cells expressing the human GCGR using polyethylenimine (Sigma-Aldrich, St. Louis, MO) (33). The cells were transfected with pCMV6-AC-RAMP2 (containing a neomycin resistance gene) and nine nitrogen equivalents of polyethylenimine. Forty-eight hours later, media were supplemented with 800 μg/mL Geneticin (Thermo Fisher Scientific, Waltham, MA) to select cells containing the construct.

To establish a second independent cell line stably expressing RAMP2, CHO-K1 cells expressing the human GCGR were cotransfected with C-terminally cyan fluorescent protein (CFP)–tagged RAMP2 (Tebu-bio Ltd., United Kingdom) and a plasmid conferring puromycin resistance using lipofectamine 2000 (Thermo Fisher). Forty-eight hours later, media were supplemented with puromycin 10 μg/mL to select cells containing the construct.

### Confirmation of gene expression

RNA was extracted from cells by using a Purelink RNA Mini Kit and DNase set (Invitrogen, United Kingdom), reverse transcribed by using the High Capacity cDNA Reverse Transcription Kit (Applied Biosystems, United Kingdom), and complementary DNA amplified by qPCR (probe Hs00359352_m1) (Life Technologies, United Kingdom) via a 7900HT Fast Real-Time PCR System (Applied Biosystems).

### Whole cell binding assays

Cells were grown up to 70% confluence and resuspended in 1.5 mL assay buffer (25 mM HEPES [pH, 7.4], 2 mM MgCl_2_, 1% bovine serum albumin, 0.05% [weight-to-volume ratio] Tween 20, 0.1 mM diprotin A, and 0.2 mM phenylmethane sulfonyl fluoride). Fifty microliters of I^125^-glucagon dissolved in assay buffer at 1000 cps (final concentration, 5.6 nM), unlabeled peptide made up in 400 µL of assay buffer, and 50 µL of the cell suspension was added to each microtube, vortexed, and incubated at room temperature for 90 minutes. Microtubes were then centrifuged (15781*g*, 4°C, 3 minutes), supernatant was removed, 500 µL of assay buffer was added; the microtubes were then recentrifuged. The supernatant was again discarded and the pellets measured for *γ* radiation for 240 seconds (Gamma Counter NE1600, NE Technology Ltd., United Kingdom). The specific binding (maximal specific binding minus the nonspecific binding) was calculated for each cell line. The binding data were normalized so that the maximal specific binding (*i.e.,* when no unlabeled peptide was present) was 100%. The percentage specific binding was calculated for each peptide concentration as a percentage of the specific binding. The half-maximal inhibition concentrations (IC_50_), a measure of binding affinity, were then calculated and compared for CHO-K1-GCGR and CHO-K1-GCGR-RAMP2 cells. IC_50_ values were calculated by using GraphPad Prism 5.01 software (GraphPad Software Inc.) with the following regression fit line:

Y=Bottom+(Top-Bottom)/(1+10^((LogEC50-X)))

Where Y = percentage specific binding and X = concentration of the agonist.

To calculate receptor density (Bmax), binding data were normalized to protein content of the cell samples, as determined by a bicinchoninic acid assay (Sigma-Aldrich). Bmax was then calculated for using GraphPad Prism 7.0b (GraphPad Software Inc., USA) using the following regression fit line:

Y=(Bmax∗HotnM)/(HotnM+ColdNM+KdNM)+Bottom

### Cell surface expression experiment

CHO-K1 cells overexpressing the human GCGR (with or without RAMP2) were seeded overnight in 96-well plates (30,000/well). Following fixation (2% paraformaldehyde), an in-cell enzyme-linked immunosorbent assay (ELISA) was performed in nonpermeabilized cells to detect surface GCGR expression. Antibodies used were rabbit primary vs GCGR *N*-terminus (1:200, ab137649; Abcam, United Kingdom) and anti-rabbit IgG horseradish peroxidase–conjugated secondary (1:2000, #15015; Active Motif, United Kingdom), with 2% bovine serum albumin block used during in all incubations. 3,3′,5,5′-Tetramethylbenzidine substrate (Thermo Fisher Scientific, United Kingdom) was added and absorbance read at 450 nm after addition of 1 M HCl. Surface GCGR expression was calculated as absorbance after subtraction of nonspecific binding (determined in the absence of primary antibody) and normalization to protein content (bicinchoninic acid assay).

### Cyclic adenosine monophosphate accumulation assay for activation of adenylyl cyclase

CHO-K1 cells overexpressing the human GCGR (with or without RAMP2), plated onto 48-well plates at 40,000 cells/well, were incubated in serum-free media for 1 hour. Peptides were prepared in serum-free DMEM containing 100 µM of IBMX (3-isobutyl-1-methylxanthine; Sigma-Aldrich, United Kingdom). The cells were incubated for 30 minutes with the test peptide, after which media were replaced with 110 µL lysis buffer (0.1M HCl with 0.5% Triton-X). The lysate was assayed by using a direct cyclic adenosine monophosphate ELISA kit (Enzo Life Sciences, United Kingdom), as described in the assay manual. The cyclic adenosine monophosphate (cAMP) response was corrected for well protein levels (Bradford reagent; Sigma-Aldrich) and expressed as a percentage of response to 10 µM forskolin.

Human hepatoma 7 cells overexpressing the human GCGR (Huh7-GCGR) were cultured in DMEM supplemented with 10% fetal bovine serum, 100 IU/mL penicillin, 100 μg/mL streptomycin, and 10 μg/mL geneticin (standard maintenance media). They were plated onto 96-well plates at 20,000 cells/well in standard maintenance media with transfection reagents for gene silencing (see details later). After 72 hours, media were aspirated and replaced with 40 µL of glucagon at different concentrations, prepared in serum-free DMEM. The cells were incubated for 30 minutes with the glucagon, after which an equal volume of cAMP lysis buffer (CisBio cAMP Dynamic cell based assay kit) was added to each well. Twenty-five microliters of lysate was transferred to a homogeneous time resolved fluorescence–compatible plate, and 12 µ: of D reagent was added to each well, followed by 12 µL of K reagent in accordance with the manufacturer’s instructions. The plate was read (i3x plate reader; Molecular Devices) after 1 hour of incubation at room temperature, and cAMP response was expressed as a percentage of response to 10 µM forskolin.

The maximal response (E_max_) and the half-maximal effective concentrations (EC_50_) were then calculated and compared for each peptide tested between CHO-K1-GCGR and CHO-K1-GCGR-RAMP2 cells, and for glucagon between Huh7-GCGR RAMP2 knockdown and Huh7-GCGR control cells. EC_50_ values were calculated by using the following regression fit line:

Y=Bottom+(Top-Bottom)/(1+10^((LogEC50-X)∗HillSlope))

where Y = cAMP response and X = agonist concentration. The curve fitting for the biphasic curves was done by using a variable slope (four parameters) model.

### Intracellular Ca^2+^ flux assay

The DiscoveRx Ca NW^PLUS^ Assay Kit (DiscoveRx Corporation Ltd., United Kingdom) was used as per the manufacturer’s protocol to detect changes in intracellular Ca^2+^ in CHO-K1 cells overexpressing the human GCGR (with or without RAMP2) in response to glucagon, GLP-1, oxyntomodulin, and analog G(X). Cells, plated overnight onto 96-well plates at 50,000 cells/well, were incubated in 75 µL Ca NW^PLUS^ working reagent for 1 hour at 37°C. Twenty-five microliters of glucagon (or related peptide) was applied from the reagent plate to the cell plate via the integrated transfer pipetter of a fluorescent microplate reader (NOVOstar; BMG Labtech Ltd., United Kingdom). Fluorescence signal was measured from 5 seconds before to 30 seconds after injection of agonist. The Ca^2+^ response was expressed as a percentage of the adenosine triphosphate response (1 µM).

### *β*-Arrestin recruitment assay

PathHunter CHO-K1 GCGR *β*-Arrestin GPCR assay (DiscoveRx Corporation Ltd.) was used to determine the effect of RAMP2 on the potency of GCGR ligands for recruitment of *β*-arrestin-1 to the GCGR. The CHO-K1-*β*Arr-GCGR cells are engineered to detect the interaction of *β*-arrestin with the activated GCGR using *β*-galactosidase fragment complementation. CHO-K1-*β*Arr-GCGR cells were stably transfected with or without RAMP2, as described previously. Cells, plated at 100 µL/well into a 96-well plate were incubated with glucagon, GLP-1, oxyntomodulin, or G(X) (10 µL) for 90 minutes at 37°C and 5% CO_2_. Fifty-five microliters of the PathHunter detection reagents was added to each well, and the microplate was incubated at room temperature for 60 minutes.

### Small interfering RNA knockdown

Small interfering RNA (siRNA) knockdown of RAMP2 in CHO-K1-GCGR-RAMP2 and CHO-K1-*β*Arr-GCGR-RAMP2 cells was performed by using pooled siRNA to RAMP2 previously validated by Albertin *et al.* (34). The siRNA complexes (fully deprotected and desalted; Sigma-Aldrich), added in a single pool (containing four duplexes) at final concentrations of 10 nM and 50 nM, were used for transfection with siPORT NeoFX (Ambion). siPORT NeoFX (diluted 1:20 into serum-free medium) and RNAs were combined (1:1) and incubated for 10 minutes at room temperature. The complexes (200 µL/well) were then dispensed into a 6-well plate and 2.3 mL of cell suspension containing 150,000 cells/well was added. The effects on *RAMP2* gene expression were assessed 24 hours later. The effect of RAMP2 knockdown on GCGR signaling was carried out in a 96-well plate 24 hours later, with volumes adjusted as follows: siRNA, 10 µL/well; SiPORT NeoFX, 10 µL/well; cell suspension, 80 µL (6000 cells)/well.

In Huh7-GCGR cells, RAMP2 expression was transiently silenced by using siRNA against human RAMP2 (Ramp2 Silencer Select siRNA; Ambion). Lipofectamine 2000 reagent (Thermo Fisher Scientific) was diluted in Opti-MEM Reduced Serum medium (Thermo Fisher Scientific) (0.2 µL/5 µL) and then added to siRNA also diluted in Opti-MEM (0.5 pmol/5 µL) for an incubation period of 5 minutes. The siRNA–lipofectamine complex (final volume, 10 µL/well) was dispensed into the wells of a 96-well plate, and to each well 100 µL of cell suspension at 150,000 cells/well was added. Cells were incubated for 72 hours. Control cells underwent exactly the same procedure except with siRNA with no gene target (Silencer Select Negative Control No.1 siRNA; Thermo Fisher Scientific).

### Confocal microscopy

HEK293 cells were stably transfected with C-terminal green fluorescent protein (GFP)–tagged GCGR (Origene) using Lipofectamine 2000 (Life Technologies Ltd., United Kingdom) as per the manufacturer’s protocol. GFP-tagged GCGR-expressing HEK293 cells were seeded onto sterile coverslips coated with poly-l-lysine in a 6-well plate and transiently transfected ±C-terminally CFP-tagged RAMP2 (Tebu-bio Ltd., United Kingdom), nontagged RAMP2 (Origene), or empty vector (pcDNA3.1). The following day, cells were fixed with 2% paraformaldehyde (Sigma-Aldrich) and mounted with Vectashield (Vector Laboratories Ltd., United Kingdom). A first set of experiments was carried out by using an X-Light spinning disk system (Crest Optics) coupled to an Eclipse Ti microscope (Nikon) and a 63× 1.4 numeric aperture oil immersion objective. GFP was excited by using a solid-state at *λ* = 491 nm laser (cobalt) and emitted signals collected at *λ* = 525/25 nm using a highly sensitive Orca-Flash4.0 Digital CMOS camera (Hamamatsu). Because of bleed-through of the intense GFP signal into the CFP channel at *λ* = 440 nm, the latter fluorophore was instead excited slightly off-peak by using a solid-state 405-nm laser and emitted signals were collected at *λ* = 525/25 nm. A second set of experiments was performed by using a Zeiss LSM780 confocal microscope and a 63× 1.2 numeric aperture water immersion objective. GFP and CFP were excited by using a *λ* = 488 nm argon laser and emitted signals were collected at *λ* = 510 to 550 nm by using a gallium arsenide phosphide spectral detector. CFP was excited by using a *λ* = 405 nm diode laser and emitted signals were collected at *λ* = 455-490. Images were postprocessed by using Zen software (Zeiss, United Kingdom) and subjected to Gaussian smoothing (1.3) to remove noise. Uniform linear adjustments were applied to contrast and brightness in order to improve image quality for analysis and presentation purposes, while preserving the pixel dynamic range and the intersample intensity differences. Cell surface expression of GCGR-GFP was calculated by using the threshold plugin for ImageJ software (National Institutes of Health, Bethesda, MD).

### Statistical analysis

**Figure 1.**
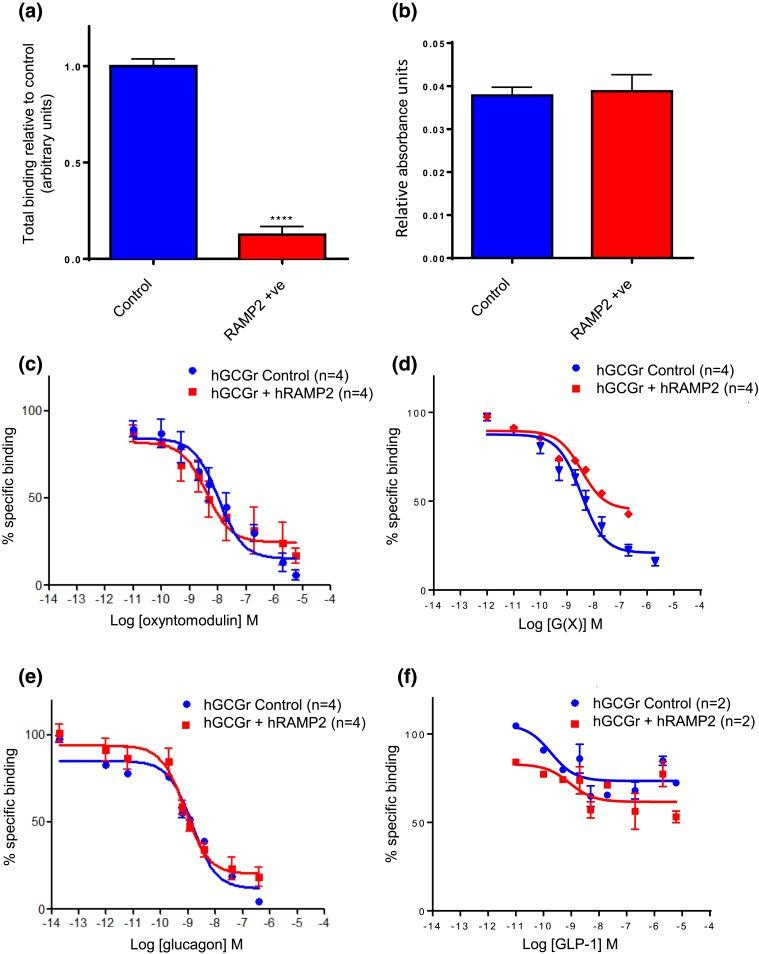
(a) Specific binding of I^125^-glucagon to the GCGR in CHO-K1-GCGR cells with or without RAMP2 (*P* < 0.0001). (b) The protein content was determined by Bradford assay (used here as a surrogate marker for the number of cells) for CHO-K1-GCGR cells with or without RAMP2. Whole cell binding of (c) glucagon, (d) GLP-1, (e) oxyntomodulin, and (f) analog G(X) to the human GCGR (hGCGR). Whole CHO-K1-GCGR cells with or without RAMP2 were used. I^125^-glucagon was used as the competing peptide in all assays and IC_50_ values were calculated as a mean of four separate experiments (except for GLP-1, for which n = 2), with each peptide concentration performed in duplicate or triplicate during an individual experiment. Values represent the mean ± standard error of the mean. hRAMP2, human RAMP2.

**Figure 2.**
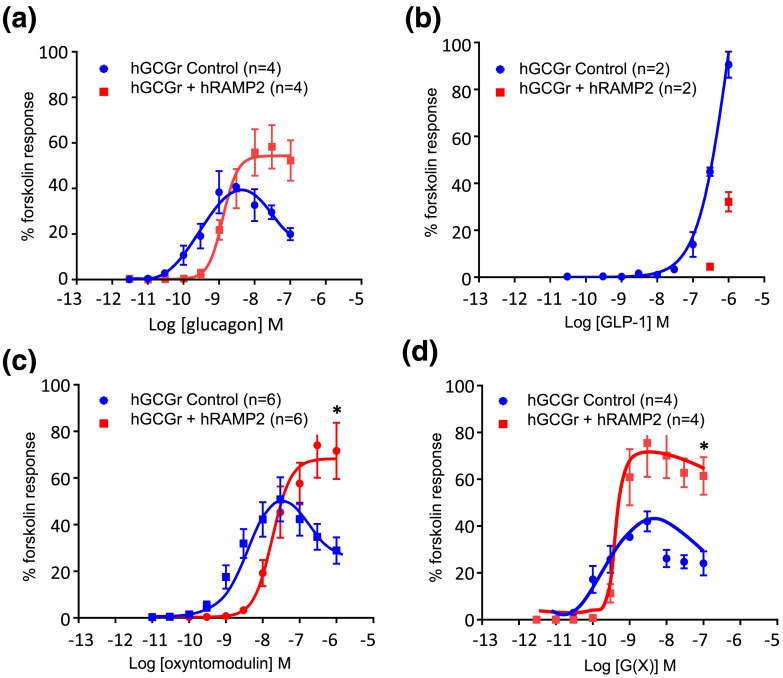
Human GCGR (hGCGR)–mediated cAMP accumulation in CHO-K1-GCGR cells with or without RAMP2 by ligands (a) glucagon, (b) GLP-1, (c) oxyntomodulin and (d) analog G(X). Each peptide concentration was tested in duplicate or triplicate in each experiment. Values calculated as a mean from a minimum of four separate experiments (unless stated otherwise). *P < 0.05 comparing E_max_ for CHO-K1-GCGR cells with or without RAMP2. Values represent the mean ± standard error of the mean. hRAMP2, human RAMP2.

**Figure 3.**
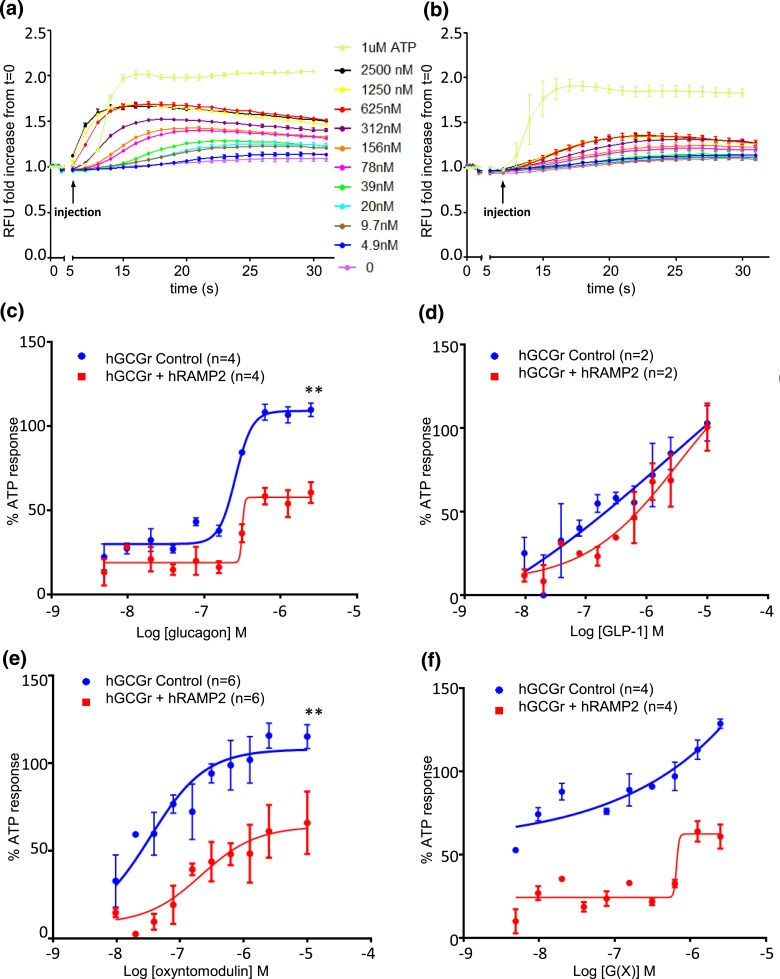
Intracellular Ca^2+^ flux in response to varying doses of glucagon in real time in CHO-K1-GCGR cells (a) without RAMP2 and (b) with RAMP2 [measured in relative fluorescence unit (RFU) fold increase from baseline RFU]. Human GCGR (hGCGR)–mediated Ca^2+^ flux in CHO-K1-GCGR cells with or without RAMP2 by ligands (c) glucagon, (d) GLP-1, and (e) oxyntomodulin and (f) analog G(X). Each peptide concentration was tested in duplicate or triplicate in each experiment. Values calculated as a mean from a minimum of four separate experiments (unless stated otherwise). **P < 0.01 comparing E_max_ for CHO-K1-GCGR cells with or without RAMP2. Values represent the mean ± standard error of the mean. ATP, adenosine triphosphate; hRAMP2, human RAMP2.

**Figure 4.**
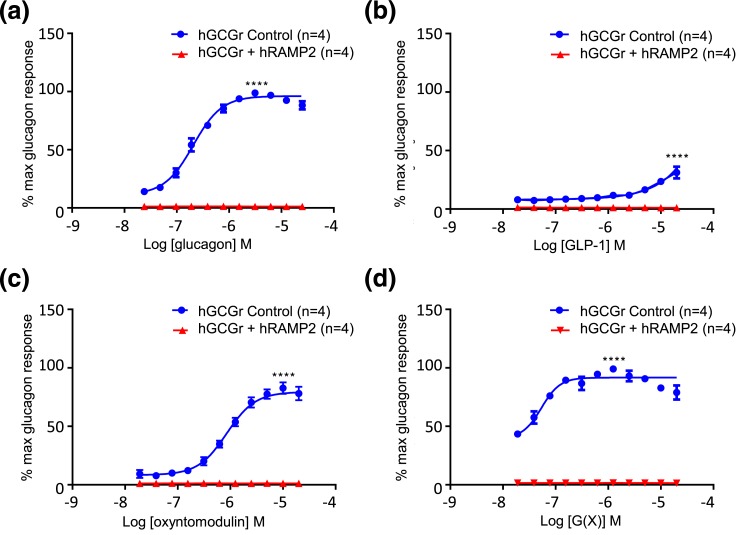
Human GCGR (hGCGR)–mediated *β*-arrestin recruitment in CHO-K1- *β*Arr-GCGR cells with or without RAMP2 by endogenous ligands (a) glucagon, (b) GLP-1, and (c) oxyntomodulin and (d) analog G(X). Each peptide concentration was tested in duplicate or triplicate in each experiment. Results are expressed as a percentage of maximal glucagon-mediated *β*-arrestin recruitment. Values calculated as a mean from a minimum of four separate experiments. ****P < 0.0001 comparing E_max_ for CHO-K1-*β*Arr-GCGR cells with or without RAMP2. Values represent the mean ± standard error of the mean. hRAMP2, human RAMP2.

**Figure 5.**
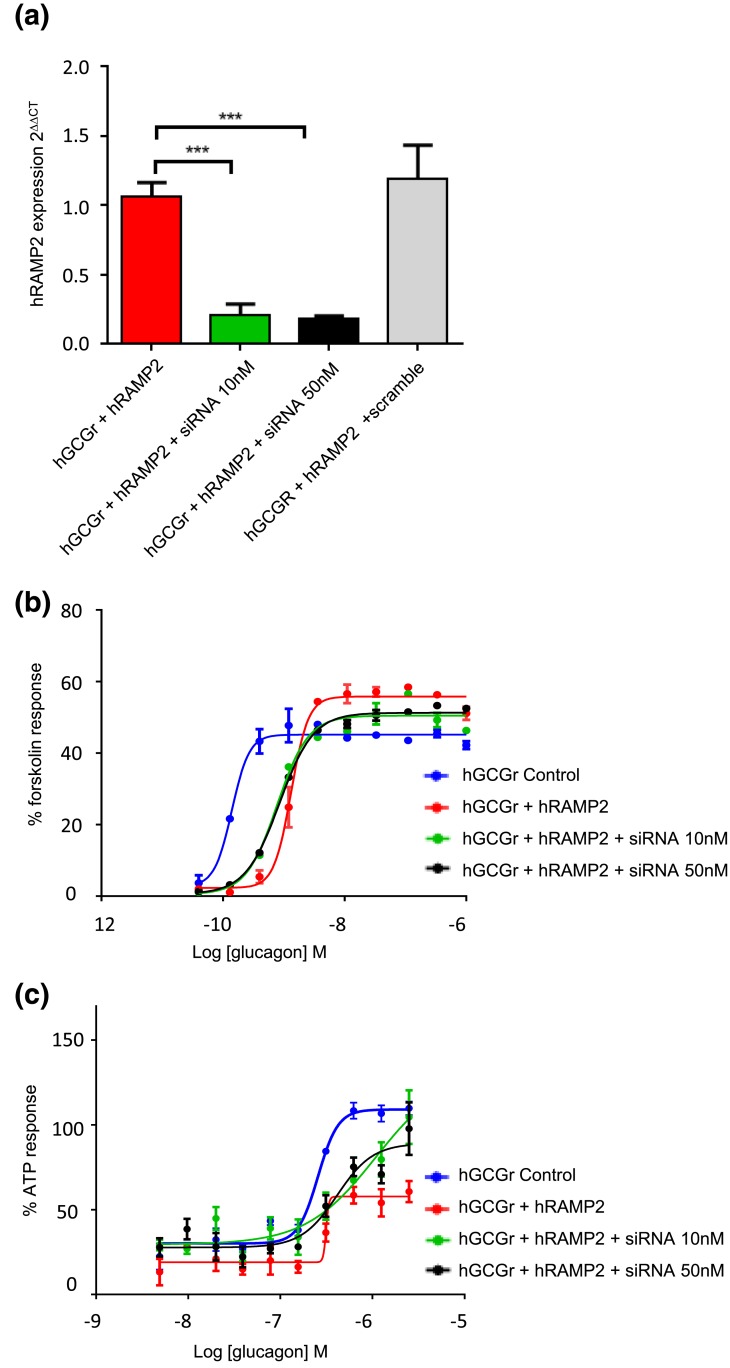
Effect of siRNA knockdown of RAMP2 in CHO-K1-GCGR-RAMP2 cells on (a) human RAMP2 (hRAMP2) expression by qPCR, (b) cAMP accumulation, and (c) Ca^2+^ flux in response to glucagon. Each peptide concentration was tested in duplicate or triplicate in each experiment. Values calculated as a mean from a minimum of two separate experiments. **P < 0.01; ***P < 0.001. Values shown are ±standard error of the mean. ATP, adenosine triphosphate.

E_max_ and EC_50_ values, derived through 4-parameter curve fit, were compared by paired *t* test. Prism software, version 5.01 (GraphPad Software Inc.), was used for statistical analysis. *P* < 0.05 was conventionally considered to indicate a statistically significant difference. Zero concentration points were not included on the graphs in Figs. 1–5 for ease of viewing. RAMP2 expression were compared between two groups by using unpaired or paired Student *t* test, or where multiple comparisons were required, one-way analysis of variance followed by Bonferroni multiple-comparisons *post hoc* test. Controls with no peptide added were included in all experiments.

## Results

### Confirmation of transfection of CHO-K1-GCGR and CHO-K1-*β*Arr-GCGR cells with RAMP2

Successful transfection into CHO-K1-GCGR cells of the pCMV6-AC-RAMP2 plasmid was confirmed by qPCR. Subsequently, a second CHO-K1 cell line expressing the GCGR containing the *β*-arrestin recruitment reporter signal (CHO-K1-*β*Arr-GCGR) was also transfected with RAMP2 [Supplemental Fig. 1(A) and (B)].

Similarly, successful transfection of CHO-K1-GCGR cells with C-terminally CFP-tagged RAMP2 (Tebu-bio Ltd.) was confirmed by qPCR. RAMP2 was undetectable in the control cell line but expressed in the CHO-K1-GCGR-CFP-RAMP2 cells.

### RAMP2 reduces specific glucagon binding at the GCGR

When specific glucagon binding to the GCGR was compared in RAMP2-positive and -negative CHO-K1 cells, it was found to be 10-fold lower in the presence of RAMP2 [Fig. 1(a)]. This was despite the protein content being similar in both groups [Fig. 1(b)].

Glucagon bound to the GCGR with an IC_50_ of 1.403 nM. This was not significantly altered when the GCGR was coexpressed with RAMP2 [Fig. 1(c) and Table 1). As expected, GLP-1 had poor affinity for the GCGR, with an IC_50_ of >10,000 nM [Fig. 1(d)]. Oxyntomodulin and analog G(X) showed a 7-fold and 2.5-fold lower affinity for the GCGR than the native peptide, respectively [Fig. 2(e) and 2(f)]. Similar to glucagon, the presence of RAMP2 had no effect on the binding affinity at the GCGR for GLP-1, oxyntomodulin, or analog G(X).

**Table 1.** **Binding Affinities of Glucagon, GLP-1, Oxyntomodulin, and Analog G(X) to the Human GCGR**

**Variable**	**IC_50_**	
	GCGR Control (nM)	GCGR + RAMP2 (nM)
Glucagon	1.403 ± 0.21	0.768 ± 0.15
GLP-1	>10,000	>10,000
Oxyntomodulin	10.43 ± 2.59	3.873 ± 0.93
G(X)	3.381 ±1.07	3.984 ± 1.81

Whole CHO-K1-GCGR cells with or without RAMP2 were used. I^125^-glucagon was used as the competing peptide in all assays and IC_50_ values were calculated as a mean of four separate experiments (except for GLP-1, for which n = 2), with each peptide concentration performed in duplicate or triplicate during an individual experiment. Errors shown are ±standard error of the mean.

To ensure that these findings were attributable to coexpression of RAMP2 with the GCGR, a second independent cell line with RAMP2 stably upregulated was investigated (CHO-K1-GCGR-CFP-RAMP2) and compared with a cell line transfected in parallel with a control (pcDNA3.1) plasmid. As with the first cell line (CHO-K1-GCGR-RAMP2), the binding affinity of glucagon for its receptor was not altered with the upregulation of RAMP2 (IC_50_, 4.377 nM with CFP-RAMP2 vs 5.123 nM without; *P* = 0.16) [Supplemental Fig. 2(A)]; however, the density of GCGR binding sites (Bmax) was significantly lower in the cell line with upregulated RAMP2 (*P* = 0.0069) [Supplemental Fig. 2(B)].

### RAMP2 reduces cell surface expression of the GCGR

By using an in-cell ELISA, surface GCGR expression was detected in nonpermeabilized CHO-K1-GCGR cells (with or without RAMP2) (Supplemental Fig. 3). GCGR cell surface expression was significantly reduced in cells expressing RAMP2.

### RAMP2 reduces potency and increases efficacy of the G*α*s pathway at the GCGR

To assess whether RAMP2 affected the G*α*s pathway, cAMP accumulation was measured in its presence/absence in CHO-K1 cells (Fig. 2 and Table 2). In control cells, the highest concentrations of peptide resulted in cAMP accumulation lower than the E_max_, which is a well-described desensitization effect (14). In the presence of RAMP2, glucagon, oxyntomodulin, and analog G(X) increased the EC_50_ (*i.e.*, RAMP2 reduced the potency of these ligands for GCGR) [Fig. 2(a), 2(c), and 2(d)]. When the GCGR was stimulated by oxyntomodulin or analog G(X), the E_max_ (efficacy) was increased in the presence of RAMP2. The EC_50_ and E_max_ were not calculable for GLP-1 response at the concentrations used [Fig. 2(b)]. There was no significant difference in cAMP responses to forskolin between control and RAMP2-expressing cells [0.136 ± 0.01 vs 0.140 ± 0.01 (mean ± standard error of the mean) relative absorbance units, respectively; *P* = 0.20].

**Table 2.** **Summary of cAMP Accumulation and Ca^2+^ Data for Glucagon, GLP-1, Oxyntomodulin and Analog G(X) at the GCGR**

**Variable**	**cAMP Accumulation**		**Intracellular Ca^2+^ Flux**	
	CHO-K1-GCGR Cells	CHO-K1-GCGR Cells + RAMP2	CHO-K1-GCGR Cells	CHO-K1-GCGR Cells + RAMP2
Glucagon				
EC_50_ (nM)	0.161 ± 0.063	1.263 ± 0.289*^a^*	256.5 ± 27.46	314.1 ± 37.03
E_max_ (%)	34.04 ± 6.897	54.50 ± 9.781	109.0 ± 2.215	57.7 ± 1.313*^b^*
GLP				
EC_50_ (nM)	NA	NA	NA	NA
E_max_ (%)	NA	NA	NA	NA
Oxyntomodulin				
EC_50_ (nM)	1.089 ± 0.382	12.97 ± 8.544*^a^*	109.6 ± 11.5	156.8 ± 43.21
E_max_ (%)	34.69 ± 6.815	46.23 ± 7.409*^a^*	108 ± 9.28	64 ± 12.3*^b^*
Analog G(X)				
EC_50_ (nM)	0.074 ± 0.056	0.538 ± 0.065*^a^*	NA	656.6 ± 35.0
E_max_ (%)	31.11 ± 3.578	65.43 ± 7.027*^a^*	NA	62.5 ± 6.06

EC_50_ is defined as the concentration of agonist required to cause 50% of the maximal possible effect of that agonist. E_max_ is the maximal response of the agonist expressed as a percentage of maximal positive control response. Values calculated as a mean from a minimum of four separate experiments (except for GLP-1, for which n = 2). Values shown as mean ± standard error of the mean.

Abbreviation: NA, not available.

^a^ *P* < 0.05 comparing CHO-K1-GCGR cells with or without RAMP2.

^b^ *P* < 0.01 comparing CHO-K1-GCGR cells with or without RAMP2.

To investigate whether changes in cAMP accumulation at the GCGR conferred by RAMP2 were generalizable to other cell types, cAMP accumulation in response to glucagon was measured in Huh7-GCGR cells with or without RAMP2 knockdown. Huh7-GCGR cells express a low level of endogenous RAMP2, and silencing conferred approximately 70% knockdown. There was no statistically significant change in glucagon potency in Huh7-GCGR cells with RAMP2 knockdown and a trend toward a lower E_max_ was seen, although this was not statistically significant (Supplemental Fig. 4).

### RAMP2 reduces efficacy of the G*α*q pathway at the GCGR

To assess the effect of RAMP2 on the G*α*q pathway, intracellular Ca^2+^ flux was measured in real time in CHO-K1 cells. For glucagon and oxyntomodulin, the Ca^2+^ response was attenuated when cells expressing the glucagon receptor were coexpressed with RAMP2, as demonstrated by a significantly lower E_max_ [Fig. 3(a–c) and 3(e)]. RAMP2 also appeared to lower the response to G(X), however, as the maximal Ca^2+^ response was not achieved with cells expressing GCGR alone and E_max_ could not be determined [Fig. 3(f)]. Similarly, the EC_50_ and E_max_ were not calculable for GLP-1 response at the concentrations used [Fig. 3(d)]. EC_50_ was unchanged in the presence of RAMP2 for all ligands (Table 2). There was no significant difference in Ca^2+^ responses to ATP between control and RAMP2-expressing cells [relative fluorescence unit fold increase from baseline, 1.81 ± 0.08 vs 1.84 ± 0.10 (mean ± standard error of the mean), respectively; *P* = 0.77] [Fig. 3(a) and 3(b)].

### RAMP2 abolishes *β*-arrestin recruitment at the GCGR

For all ligands [glucagon, GLP-1, oxyntomodulin, and analog G(X)], *β*-arrestin recruitment did not occur in CHO-K1 cells expressing both GCGR and RAMP2 (Fig. 4).

### RAMP2 knockdown partially restores GCGR functioning for the G*α*s and G*α*q pathways

Efficient siRNA knockdown of RAMP2 was achieved with both 10 nM and 50 nM siRNA pools [Fig. 5(a)]. siRNA knockdown of RAMP2 in CHO-K1-GCGR-RAMP2 cells resulted in a trend toward restoration of cAMP EC_50_ and E_max_ to levels seen with control cells (CHO-K1-GCGR cells); however, they were not significantly different from control or RAMP2 (without siRNA) cells [Fig. 5(b)]. A similar finding was demonstrated for Ca^2+^ fluxes [Fig. 5(c)]. The EC_50_ and E_max_ data are summarized in Table 3.

**Table 3.** **Effect of siRNA Knockdown of RAMP2 in CHO-K1-GCGR-RAMP2 Cells on cAMP Accumulation and Ca^2+^ Flux for Glucagon at the GCGR**

**Variable**	**CHO-K1-GCGR Cells**	**CHO-K1-GCGR Cells + RAMP2**	**CHO-K1-GCGR Cells + RAMP2** **SiRNA 10 nM**	**CHO-K1-GCGR Cells + RAMP2 SiRNA 50 nM**
cAMP accumulation				
EC_50_ (nM)	0.161 ± 0.063	1.263 ± 0.289*^a^*	0.778 ± 0.018	0.846 ± 0.018
E_max_ (%)	34.04 ± 6.897	54.50 ± 9.781	50.46 ± 0.96	51.26 ± 0.11
intracellular Ca^2+^ flux				
EC_50_ (nM)	256.5 ± 27.46	314.1 ± 37.03	387.5 ± 108.5	295.3 ± 54.2
E_max_ (%)	109.0 ± 2.215	57.7 ± 1.313*^b^*	74.13 ± 2.5	70.23 ± 1.5

EC_50_ is defined as the concentration of agonist required to cause 50% of the maximal possible effect of that agonist. E_max_ is the maximal response of the agonist expressed as a percentage of maximal positive control response. EC_50_ and E_max_ values for siRNA-treated cells were not significantly different from control or RAMP2-positive cells. Values calculated as a mean from a minimum of two separate experiments. Errors shown are ±standard error of the mean.

^a^ *P* < 0.05 comparing CHO-K1-GCGR cells with or without RAMP2.

^b^ *P* < 0.01 comparing CHO-K1-GCGR cells with or without RAMP2.

### GCGR and RAMP2 colocalize and GCGR is internalized in the presence of RAMP2

**Figure 6.**
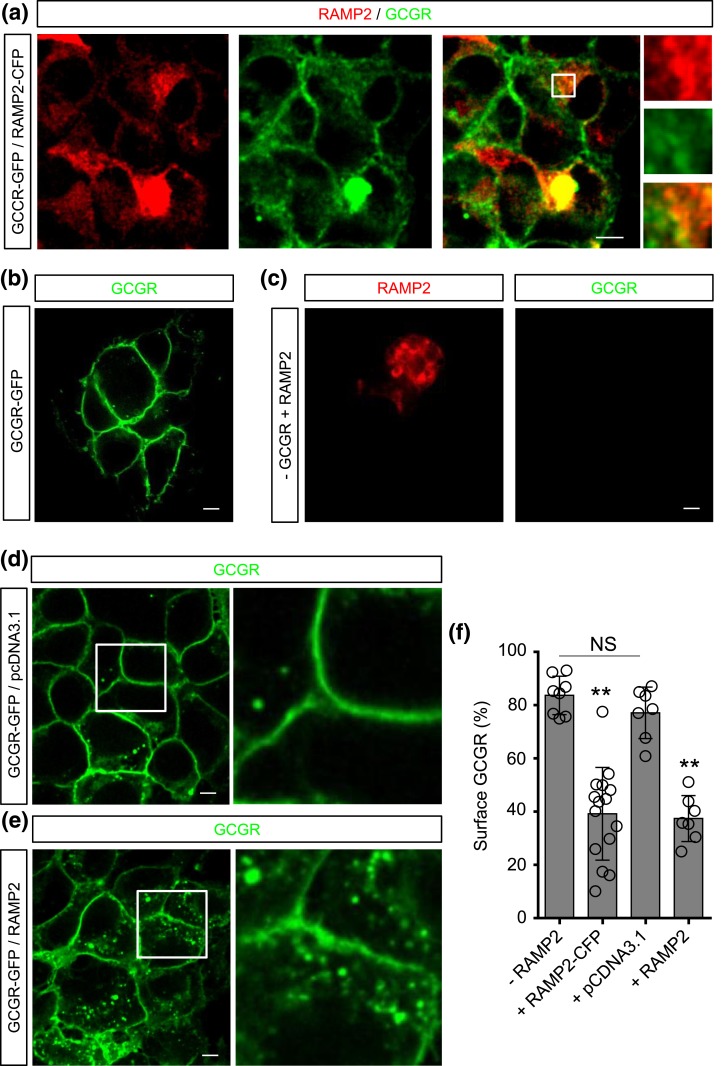
Effect of RAMP2 on GCGR localization. (a) Representative image showing that GCGR-GFP (green) and RAMP2-CFP (red) can colocalize (yellow) within the cytosolic compartment following their overexpression (n = 15 cells) (zoomed images are inset to the right). (b) Representative image showing that GCGR-GFP expression is predominantly at the membrane/surface in HEK cells without RAMP2 overexpression (n = 8 cells). (c) A negative control indicating that the 405-nm laser does not excite GCGR-GFP (n = 3 cells). (d) Overexpression of nonnative protein (pcDNA3.1) does not interfere with the distribution of the GCGR-GFP, which remains at the membrane (n = 7 cells). (e) Overexpression of nontagged RAMP2 leads to redistribution of GCGR-GFP into the cell (n = 7 cells) (zoomed images are inset to the right). (f) Bar graph showing that overexpression of either RAMP2-CFP or nontagged RAMP2, but not pcDNA3.1, leads to a significant reduction in cell surface GCGR-GFP expression (**P < 0.01) (n = 8 to 14 cells from at least three independent experiments). Scale bar = 10 µm. Values represent the mean ± standard error of the mean. NS, not significant.

High-resolution confocal microscopy showed that GCGR-GFP and RAMP2-CFP colocalized as puncta within the cytosol of HEK293 [Fig. 6(a)]. In cells where RAMP2 was not overexpressed, GCGR-GFP remained predominantly at the cell surface/membrane [Fig. 6(b)]. This was not due to bleed-through of GCGR-GFP fluorescence into the RAMP2-CFP channel because signal could not be detected in RAMP2-negative/GCGR-positive cells [Fig. 6(c)]. Overexpression of nonnative protein (pcDNA3.1) did not interfere with the distribution of the GCGR-GFP, which remained almost exclusively at the membrane [Fig. 6(d)], whereas nontagged RAMP2 led to a significant decrease in receptor at the cell membrane [Fig. 6(e)]. This demonstrates that protein expression *per se* is unlikely to interfere with GCGR localization. Thus, overexpression of RAMP2-CFP or RAMP2 consistently leads to a decrease in cell surface GCGR-GFP [Fig. 6(f)].

## Discussion

It has previously been demonstrated by immunofluorescence confocal microscopy that RAMP2 may interact with the glucagon receptor. We investigated the functional effect of this possible interaction by looking specifically at the effect of RAMP2 on (1) ligand binding at the GCGR, (2) GCGR cell signaling, and (3) GCGR subcellular distribution. Coexpression of RAMP2 with GCGR did not alter the binding affinity of glucagon or its related peptides. However, the presence of RAMP2 had a marked effect on signaling via the G*α*s and G*α*q pathways, as well as *β*-arrestin recruitment. Furthermore, RAMP2 appears to colocalize with the GCGR and influence its subcellular distribution.

Interaction between calcitonin family receptors and the individual RAMP proteins alters both ligand binding affinity and the intracellular signaling pathways engaged (17, 35, 36). By contrast, we found that expression of RAMP2 with the GCGR did not cause a significant alteration in the binding affinity of glucagon and its related peptides in whole cells. However, competition binding experiments using ^125^I-glucagon as the radioligand revealed that coexpression of RAMP2 resulted in a 10-fold reduction in GCGR binding sites when compared with those determined in the absence of RAMP. This reduction in specific binding of glucagon may be due to reduced receptor expression at the cell surface. This could have been a direct effect of the interaction of RAMP2 and the GCGR, resulting in internalization. Alternatively, it might be an indirect effect if, for example, RAMP2 influences GCGR cell surface expression via its effect on *β*-arrestin recruitment.

The presence of RAMP2 completely abolished *β*-arrestin recruitment. This finding was consistent for glucagon as well as GLP-1, oxyntomodulin, and G(X). One possible explanation is that RAMP2 interacts with the GCGR at the same site as *β*-arrestin binds or causes steric hindrance, thereby disrupting *β*-arrestin recruitment. Krilov *et al.* (13) have shown that *β*-arrestins are crucial for the recycling of the GCGR, and, therefore, loss of *β*-arrestin recruitment may result in reduced cell surface expression of the GCGR when RAMP2 is present. Alternatively, reduced cell surface expression of GCGR may be the primary effect of RAMP2, and this, in turn, may prevent *β*-arrestin recruitment.

Coexpression of RAMP2 with the GCGR also altered the intracellular signaling properties of the receptor in CHO-K1-GCGR cells, with the same effects seen for all agonists tested. With regard to the G*α*s pathway, the presence of RAMP2 caused a reduction in potency and increase in efficacy. In Huh7-GCGR cells, the knockdown of RAMP2 resulted in no change in potency and a trend toward decreased efficacy. Whether this is a result of a change in availability of binding sites is yet to be determined. In contrast to our findings, Weston *et al.* (29) found that RAMP2 increases potency of the cAMP response at the GCGR. One possible explanation for these different findings could be the different cell lines used. Weston *et al.* (29) overexpressed RAMP2 in HEK cells that already express endogenous RAMP2, whereas we overexpressed RAMP2 in CHO-K1 cells that do not express RAMP2. Previously research showed that interaction of the calcitonin receptor with RAMPs, especially RAMP2, is sensitive to the cellular background in which it is expressed, suggesting that other cellular components, such as G proteins, are likely to contribute to RAMP–receptor interactions (36).

The increase in efficacy of cAMP production observed with RAMP2 is intriguing. This enhancement in cAMP response is all the more striking as it is in the face of an apparent reduction of cell surface expression of GCGR. The simplest interpretation is that by some mechanism, RAMP2 increases the accessibility of the receptor to the G protein (37). Alternatively, RAMP2 may inhibit the desensitization response that is classically seen with the GCGR, involving phosphorylation of receptors by GPCR kinases and binding of *β*-arrestins, which uncouple receptors from G proteins (38). We speculate that the GCGR–RAMP2 interaction causes loss of desensitization, which may be driven by inhibition of *β*-arrestin recruitment. Indeed, RAMPs are crucial in the postendocytic sorting of the calcitonin receptor–like receptor, suggesting a broader regulatory role for RAMPs in receptor trafficking (24, 25).

On examination of the G*α*q pathway, intracellular Ca^2+^ fluxes were found to be attenuated in the presence of RAMP2. Interestingly, preferential coupling to G*α*s vs G*α*q has been reported for AMY1 and AMY3 receptors, but not AMY2 (39). The finding that cAMP signaling is specifically augmented and Ca^2+^ signaling attenuated by RAMP2 at the GCGR is important because the classic coupling pathway associated with GCGR activation has always been thought to be the stimulation of cAMP accumulation. Moreover, the presence or absence of endogenous RAMP2 may account for discrepancies in previous studies examining the signaling mechanisms engaged by the GCGR. Whether this is tissue-specific and dependent on the prevailing physiologic conditions is yet to be seen.

Visualization of RAMP2 and the non–ligand-bound GCGR using confocal microscopy revealed two key findings. First, RAMP2 and the GCGR showed some colocalization, although superresolution approaches are needed to confirm this, as well as delineate the compartment(s) involved. Second, in the presence of RAMP2, GCGR cell surface expression was reduced. This is consistent with the competition binding and ELISA experiments, which found reduced binding of ^125^I-GCG in the presence of RAMP2. These findings appear to be at odds with the work done by Christopoulos *et al.* (27), who reported that, when coexpressed with GCGR, RAMP2 translocates to the cell surface.

Several differences exist in the experimental approach between this current study and that of Christopoulos *et al*. (27). First, in their study only the RAMPs, and not the GCGR, were tagged, so it was not possible to comment on where the receptor was trafficked to. Second, in the Christopoulos *et al*. (27) study, RAMP2 was *N*-terminally tagged with hemagglutinin, whereas in our study both C-terminally CFP-tagged and native RAMP2 was used. It is the *N*-terminal that contains the natural, predicted signal peptide sequence of RAMP2, and therefore this may have had a bearing on expression of RAMP2. In line with our findings, using C-terminal receptor-fluorescent protein fusion constructs and cell surface ELISAs of myc-tagged receptors, Weston *et al.* (29) found that expression of RAMP2 caused a nonsignificant decrease in cell surface expression of GCGR. To ensure that the agonist-stimulated internalization response is not due to glucagon in the serum used to culture these cells, with amplification in the presence of RAMP2, further experiments could be performed with a GCGR antagonist or serum-free medium, or alternatively excess glucagon.

Taken together, this work demonstrates that RAMP2 may affect the cell signaling pathways of the GCGR as well as its trafficking within the cell. There are two possible mechanisms by which RAMP2 could influence GCGR pharmacology. A direct effect on binding epitopes of the relevant ligands is possible. Alternatively, RAMP2 could act indirectly by altering the conformation of the GCGR.

This work has added to our understanding of GCGR’s physiologic function and how this may be modified by an allosteric modulator, RAMP2. This could be important in developing new therapeutic avenues for the treatment of obesity and diabetes. Allosteric modulation through the RAMP2 system may allow "biasing" of the signaling pathways to exploit the desirable downstream effects, thus informing the construction of new peptide analogs with selective agonist activities. For example, these might incorporate therapeutically desirable properties, such as appetite suppression and increase in energy expenditure, without unwanted properties, such as increasing hepatic glucose output and hyperglycemia.

The work conducted thus far has been in GCGR-overexpressing cell lines. The logical next step would be to use primary cells in tissue relevant to glucagon receptor physiology. It would be interesting to use CRISPR-Cas9 to delete/replace the endogenous loci in a *β* or hepatocyte cell line, thus leading to stable and physiologic GCGR expression levels in the presence or absence of RAMP and study function. Additionally, endogenous tissue coexpression of RAMP2 and GCGR has not yet been investigated. RAMP messenger RNA tissue expression using Northern blot analysis was reported initially by McLatchie *et al.* (17) on their discovery of RAMPs. However, GCGR-relevant tissues, such as brown adipose tissue, hypothalamus, and the nodose ganglion, were not specifically examined. An additional question is whether the RAMP2–GCGR interaction is controlled in a physiologic setting. It would be important to determine what process controls this and what effect it has on glucagon signaling. Coexpression may occur in some tissues under certain conditions and not others because expression of RAMP2 may be controlled by the prevailing physiologic conditions, for example, glucose and insulin levels.

In conclusion, RAMP2 can affect the cell signaling pathways of the GCGR as well as its trafficking within the cell. The effect that RAMP2 has on the GCGR and how this translates *in vivo* is yet to be determined.
