# Supplementary figures and images for: RAMP2 Influences Glucagon Receptor Pharmacology via Trafficking and Signaling
Source: Endocrinology. 2017 Jun 6;158(8):2680–93. doi: 10.1210/en.2016-1755 (PMC5551549; doi:10.1210/en.2016-1755)

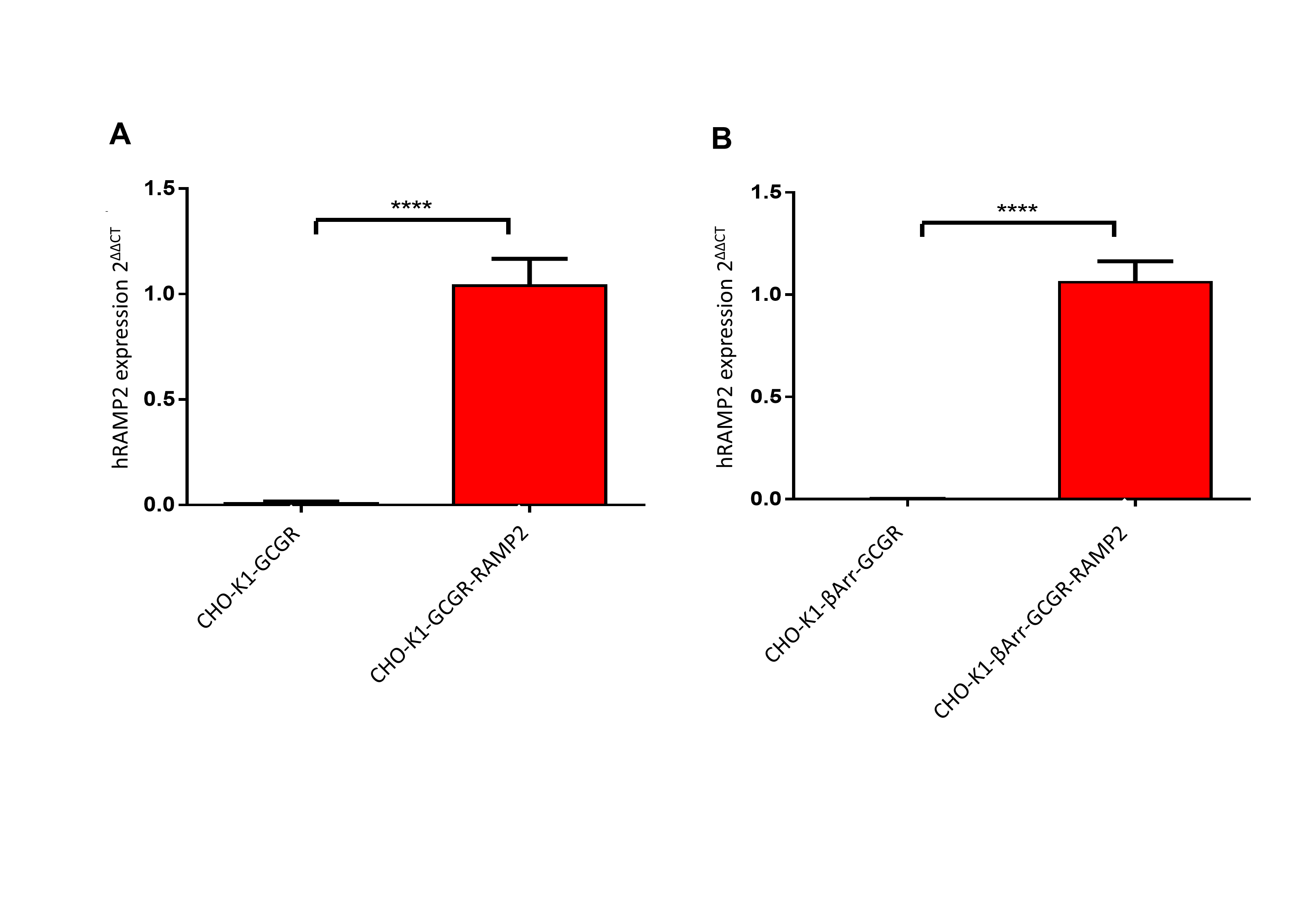

Supplement: Supplementary file 1 [file en.2016-1755.sf1.tif]

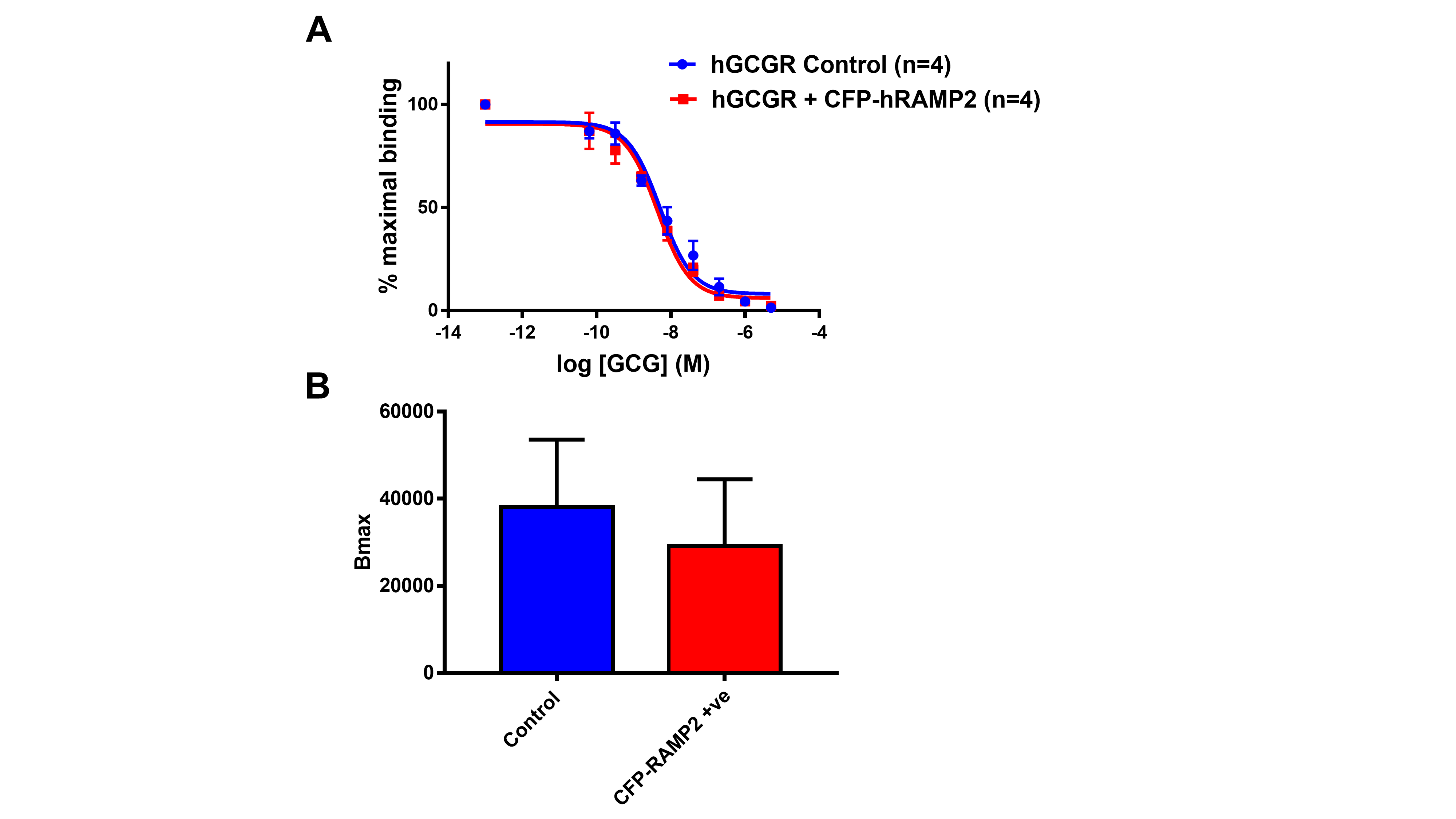

Supplement: Supplementary file 2 [file en.2016-1755.sf2.tif]

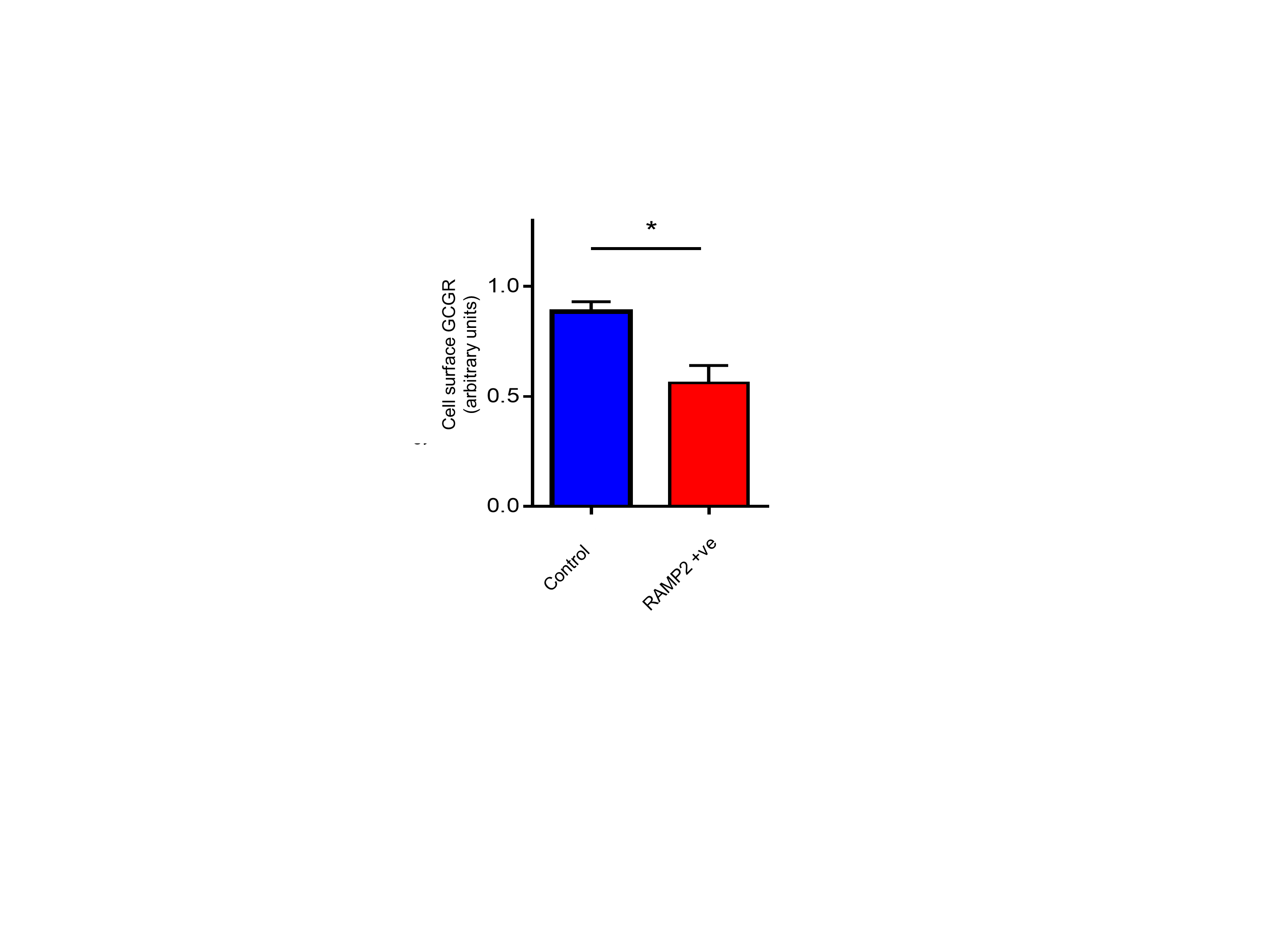

Supplement: Supplementary file 3 [file en.2016-1755.sf3.tif]

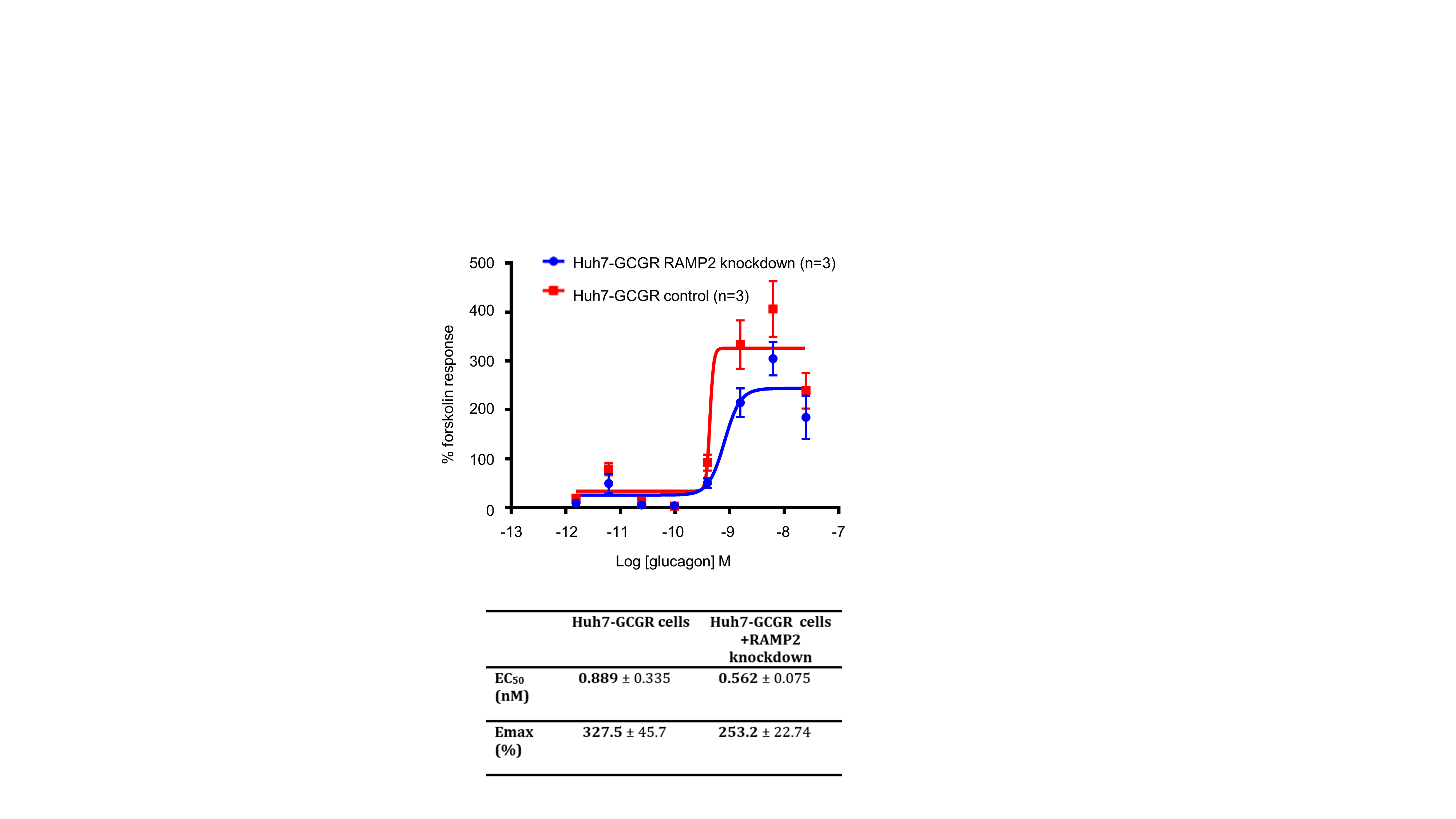

Supplement: Supplementary file 4 [file en.2016-1755.sf4.tif]
